# The Relationship Between Convergent IGH Signatures and Severity of COVID-19 Patients by Next-Generation Sequencing of B-Cell Repertoire

**DOI:** 10.3389/fmicb.2021.833054

**Published:** 2022-02-09

**Authors:** Hongliu Cai, Juan Hu, Lingtong Huang, Chunhua Gao, Mi Xu, Yuzhi Gao, Tao Sun, Xueling Fang

**Affiliations:** ^1^Department of Critical Care Units, The First Affiliated Hospital, Zhejiang University School of Medicine, Hangzhou, China; ^2^Zhejiang California International NanoSystems Institute, Zhejiang University, Hangzhou, China

**Keywords:** COVID-19, SARS-CoV-2, BCR, B-cell repertoire, next generation sequencing

## Abstract

**Object:**

To reveal convergent IGH signatures and the association with severity of coronavirus disease 2019 (COVID-19) patients.

**Method:**

A total of 25 COVID-19 inpatients were classified into three clinical conditions: mild, severe, and critical. We analyzed convergent IGH signatures by ImmuHub^®^ B-cell receptor (BCR) profiling system.

**Results:**

IGH singleton frequency in patients is significantly lower than that of healthy donors (HDs). The clonality index of IGH in patients is significantly higher than that in HDs. Nevertheless, no significant difference was observed among the three groups. The difference in IGH clonality (top five clones) between post- and pretreatment was significant in the improvement and deterioration groups. Three common public motifs were shared by all COVID-19 patients: ARDYGG, RWYFDY, and YYYYGMDV.

**Conclusion:**

B cells could recognize severe acute respiratory syndrome coronavirus 2 (SARS-CoV-2) and produce clonal expansion. Patients who had better outcomes after treatment had higher IGH clonality. Three common public motifs—ARDYGG, RWYFDY, and YYYYGMDV—might be used for vaccine development (ChiCTR2000029626).

## Introduction

Coronavirus disease 2019 (COVID-19), which has led to more than 5 million lives lost worldwide up to date, is caused by severe acute respiratory syndrome coronavirus 2 (SARS-CoV-2) infection. SARS-CoV-2 is a single-stranded positive-sense RNA virus that causes respiratory illness (Castagnoli et al., 2020; Chen et al., 2020). Several variants, including the B.1.1.7 (Alpha), B.1.351 (Beta), P.1 (Gamma), B.1.427 (Epsilon), B.1.429 (Epsilon), and B.1.617.2 (Delta), had emerged in the United Kingdom, South Africa, Brazil, and India, within several months (Voloch et al., 2021; Wilton et al., 2021).

The SARS-CoV-2 RNA has been detected in sputum, nasopharyngeal swabs, stool, and aerosol samples (Wolfel et al., 2020). Like other coronavirus infections, SARS-CoV-2 elicits a humoral response and induces T- and B-cellular immune responses (Guo et al., 2020; Sheng et al., 2021; Varchetta et al., 2021). Studies of patients’ immune systems will improve the understanding of the early-stage SARS-CoV-2 infection and formulate the relationship between disease severity and immune responses. In addition, as protective vaccines are authorized and have passed the Food and Drug Administration (FDA) approval, it is urgent to understand how the host immune system recognizes the virus and evokes the innate or adaptive immune response. To drive a deeper understanding of the nature of humoral immunity to SARS-CoV-2 infection and to identify potential therapeutic antibodies to SARS-CoV-2, we will implement the ImmuHub^®^ B-cell IGH profiling system to obtain comprehensive insights and monitor the B-cell response level from COVID-19 patients with mild to severe symptoms.

## Materials and Methods

This single-center prospective observational study was performed from January 28, 2020, to April 28, 2020, in the First Affiliated Hospital, College of Medicine, Zhejiang University. A total of 25 COVID-19 patients and 5 healthy donors (HDs) were included in the study. Patients were classified into three clinical conditions: mild, severe, and critical. *Mild group*: no symptoms or mild symptoms, like having fever or cough and other respiratory symptoms. Chest X-ray examination may display diverse pneumonia imaging characteristics or patterns. *Severe group*: patients who have one of the following symptoms: (1) shortness of breath, respiratory rate (RR) ≥ 30 times/min; (2) in the resting state, when inhaling air, the oxygen saturation ≤ 93%; (3) arterial oxygen tension (PaO_2_)/inspiratory oxygen fraction (FiO_2_) ≤ 300 mmHg; (4) clinical symptoms progressively worsened, and lung imaging showed that the lesion had progressed significantly >50% within 24 to 48 h upon chest X-ray examination. *Critical group*: patients who have one of the following symptoms: (1) respiratory failure developed, requiring mechanical ventilation; (2) shock developed; and (3) accompanied with multiple Organ failure.

This study was approved by the ethics committee of the First Affiliated Hospital, College of Medicine, Zhejiang University, and was registered in the Chinese Clinical trial registry (ChiCTR2000029626).

### The Measure of Lymphocytes

Flow cytometry (BD FACSCalibur, BD Biosciences, San Jose, CA, United States) was determined in whole peripheral blood. Cells were incubated for 30 min at 4°C with the anti-CD19 monoclonal antibodies, then centrifuged at 1,500 rpm for 5 min, and fixed with fixation buffer for 1 h. Finally, cells were washed with cell staining buffer and then resuspended in 500 μl of the same buffer for immediate flow cytometric analysis using FACSDiva software. The normal range for the counts of B lymphocytes is 86–594/μl, and that of T lymphocytes is 1,055–2,860/μl.

### ImmuHub^®^ B-Cell Receptor Profiling System

RNA extraction of peripheral blood mononuclear cells (PBMCs) was performed following RNeasy Plus Mini Kit (Qiagen, Hilden, Germany). One common forward primer adaptor and one reverse primer corresponding to the constant (C) regions of each IGH (IgG/IgA/IgM/IgD/IgE) were designed to facilitate PCR amplification of cDNA sequences in a less biased manner. Samples were analyzed by high-throughput sequencing of IGH using the ImmuHub^®^ B-cell receptor (BCR) profiling system at a deep level (ImmuQuad Biotech, Hangzhou, China) as previously described (Liu et al., 2020; He et al., 2021). Briefly, a 5′ RACE unbiased amplification protocol was used. This protocol uses unique molecular identifiers (UMIs) introduced in cDNA synthesis to control bottlenecks and eliminate PCR and sequencing errors. Sequencing was performed on an Illumina NovaSeq^®^ system with PE150 mode. During the first-strand cDNA synthesis, one common adaptor with UMI was added on the 5′ of cDNA. One reverse primer corresponding to the constant (C) regions of each IGH was designed to facilitate PCR amplification of cDNA sequences in a less biased manner. The UMI attached to each raw sequence reads was applied to correct PCR and sequencing errors and to remove PCR duplicates. V, D, J, and C segments were mapped with IMGT^®^, then complementarity-determining region 3 (CDR3) regions were extracted, and clonotype was assembled for all clones. We also utilized Multiple EM for Motif Elicitation (MEME) (Bailey et al., 2015) analysis^1^ to discover the high-frequency CDR3 motifs in the immune repertoires from COVID-19 patients. Specific sequence motifs usually mediate a common function, such as epitope-specific responses.

### Statistical Methods

Continuous variables were reported as median [interquartile ranges (IQRs)]. Categorical variables were reported as percentages. Continuous variables were compared using the Mann–Whitney U test, and the Kruskal–Wallis ANOVA compared three or more groups. Comparisons between groups for categorical variables were carried out with a chi-square (χ^2^) test. All analyses were calculated using GraphPad Prism software version 6.01. *p*-Values less than 0.05 (two-sided) were considered statistically significant.

## Results

We collected 32 PBMC samples from 25 COVID-19 patients with active infection; on the second day of admission, peripheral blood samples from all patients were collected; and again after 7 days of treatment, samples were collected from 7 critical patients.

**FIGURE 1:**
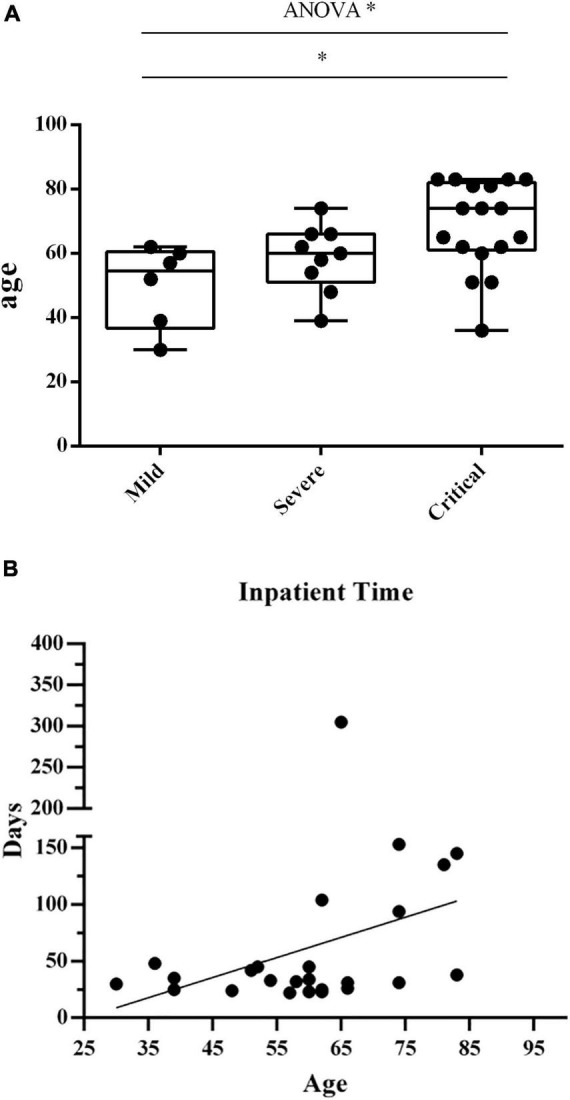
**(A)** The age in the 3 groups of patients. The age distribution in three groups of patients. Each dots indicate a single individual patient. *p < 0.05. **(B)** Correlation between patients’ age and hospitalization time. Correlation analysis between patients’ age and hospitalization time length. Each dots indicate a single individual patient. The *X*-axis shows the age of patients, and the *Y*-axis indicates the duration of hospitalization from the first visit to discharge.

The median age of the 6 patients (2 males and 4 females) in the mild group is 54 (30–62) years, that of 9 patients (7 males and 2 females) in the severe group is 60 (39–73) years, and that of 10 patients (7 males and 3 females) in the critical group is 70 (36–83) years. The median age of the 5 HDs (1 male and 4 females) is 26 (22–35) years. The age of the critical group was significantly higher than that of the mild group, *p* < 0.05 (Figure 1A). There is no significant difference in gender among the four groups.

We investigated the correlation between patients’ age and hospitalization duration (Figure 1B). We observed a positive correlation between age and hospitalization time length (*r* = 0.3921, *p* = 0.0526).

### Blood Lymphocytes

**FIGURE 2:**
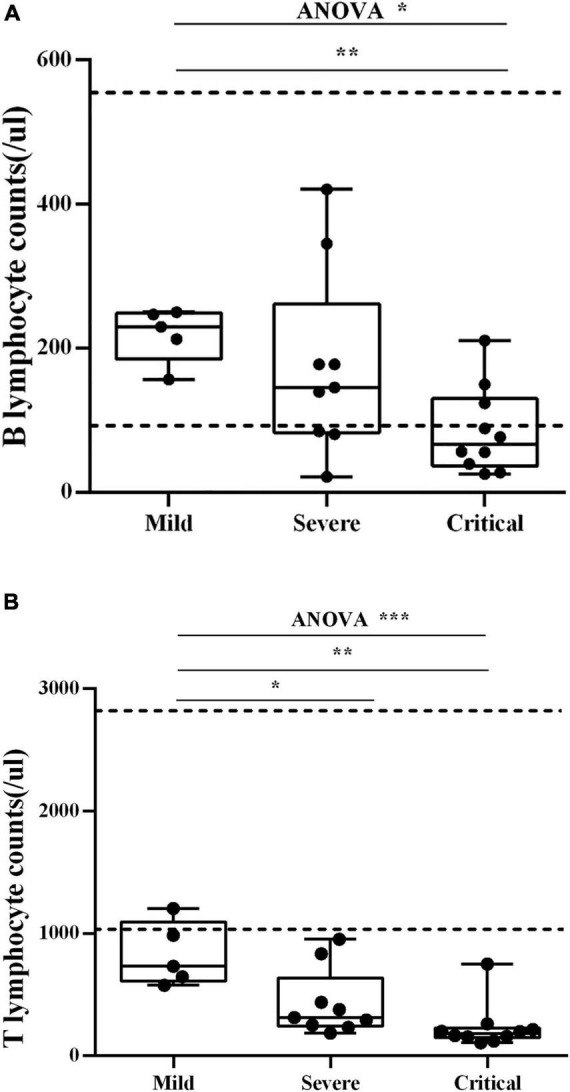
**(A)** Blood B lymphocyte counts. Blood B lymphocyte counts were measured in three groups of patients. The dash lines indicate the standard clinical range for healthy people. Each dot corresponds to a sample from a single patient. *p < 0.05, **p < 0.01. **(B)** Blood T lymphocyte counts. Blood T lymphocyte counts were measured in three groups of patients. The dash lines indicate the standard clinical range for healthy people. Each dot corresponds to a sample from a single patient. *p < 0.05, **p < 0.01, ***p < 0.001.

Blood B lymphocyte (CD19+) counts were measured in three groups of patients (Figure 2A). The results showed that B lymphocyte counts from mild and severe individuals are in the standard clinical range. When comparing the number of B lymphocytes in the three groups, the mild group was significantly higher than the critical group [median (IQR) 230 (185–248.5)/μl vs. median (IQR) 67 (37–130.5)/μl], *p* < 0.01. Blood T lymphocyte (CD3+) counts were measured in three groups of patients (Figure 2B). T lymphocyte counts of almost all individuals are below the standard clinical range. And T lymphocytes in the mild group [median (IQR) 733 (612–1,095)/μl] were significantly higher than those of the severe group [median (IQR) 314 (243–637)/μl] and the critical group [median (IQR) 183 (148–228)/μl], *p* < 0.01.

### IGH Clonality and Singleton Frequency

**FIGURE 3:**
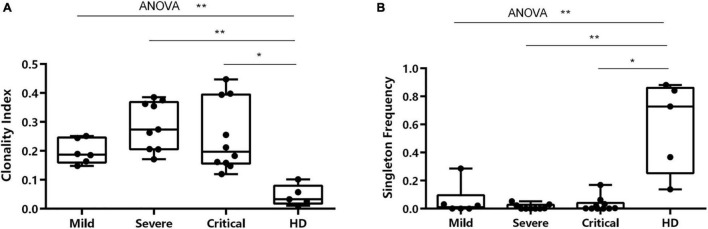
Characterization of clonality index and singleton frequency. **(A)** Characterization of clonality index in individuals across four conditions. **(B)** Differences in singleton frequency in individuals across all conditions. *p < 0.05, **p < 0.01.

Patients showed notable variations of BCR repertoire expansion and diversity. The clonality index of IGH in patients in the severe group [median (IQR) 0.2741 (0.2057–0.3685)] and critical group [median (IQR) 0.1973 (0.1560–0.3948)] was significantly higher than that of HDs [median (IQR) 0.0328 (0.0179–0.0793)] (Figure 3A). The IGH singleton frequency (the clones that only met once during sequencing) in patients in the severe group [median (IQR) 0.0006 (0.0002–0.0273)] and critical group [median (IQR) 0.0027 (0.0009–0.0401)] was significantly lower than that of HDs [median (IQR) 0.7278 (0.2527–0.8613)] (Figure 3B). Nevertheless, no significant difference was observed among the three groups.

### IGH Isotype Characteristics Among Groups

**FIGURE 4:**
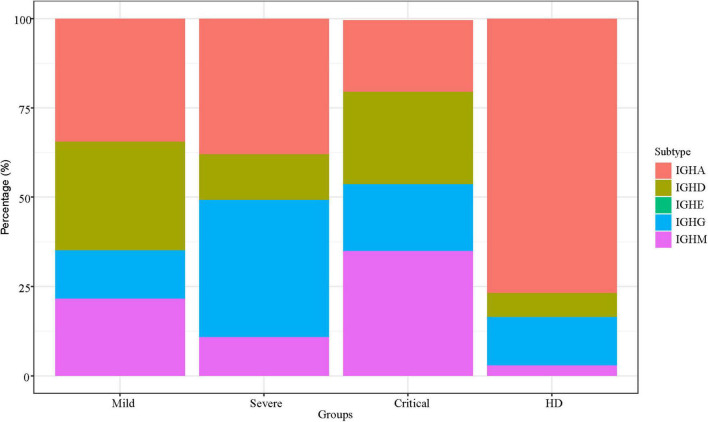
IGH isotype characteristics. IGH isotype characteristics of SARS-CoV-2 infection across patients. The *Y*-axis shows the percentage of each subtype of IGH.

We analyzed the IgA, IgD, IgE, IgG, and IgM in all SARS-CoV-2-infected patients with different clinical severity. The percentage of IGH isotypes among the three groups was summarized into stacked bar plots (Figure 4). IgA of HDs was higher than that of the critical group, *p* < 0.05. IgA and IgG of patients with critical illness were lower than those of the other two groups, and IgM was higher than the other two groups but not significantly different from that of the other group.

### Clonality Expansion

**FIGURE 5:**
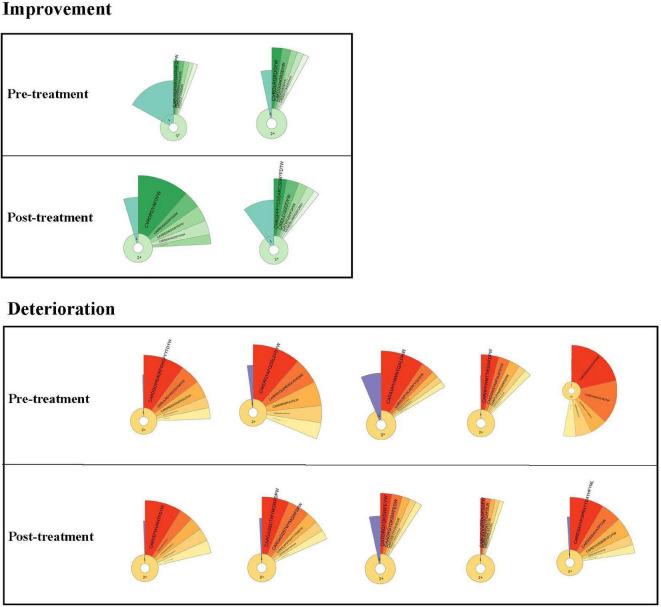
Donut charts of IGH. The donut charts of seven patients for post- and pretreatment. The fan-shaped area depicts the corresponding clonal frequency. The area of “1” in the donut chart represents the frequency of singletons. “2+ ” area represents the sequences whose reads were two or more than two. A larger radian means a higher clonal frequency. The top five clones’ amino acid sequences of complementarity-determining region 3 (CDR3) of IGH are shown.

We evaluated the dynamics in IGH repertoires and therapeutic effects in seven critical patients. The features of clonality and amino acid sequences of the top five IGH clones are shown by donut charts (Figure 5). The seven individuals were divided into the improvement group and the deterioration group. After 7 days of treatment, the fan-shaped area showed a larger radian on the donut charts in the improvement group, and the top five clones’ total frequency increased. However, the radian area of the top five clones was diminished in the deterioration group.

To better compare the total frequency of the top five clones’ dynamic changes in two groups, we summarized the IGH clonality changes between post- and pretreatment. Data of IGH clonality showed that the improvement group [median (IQR) 0.1051 (0.0279–0.1824)] was significantly higher than the deterioration group [median (IQR) −0.0739 (−0.2163 to −0.0516)].

### COVID-19-Associated IGH Motifs

**FIGURE 6:**
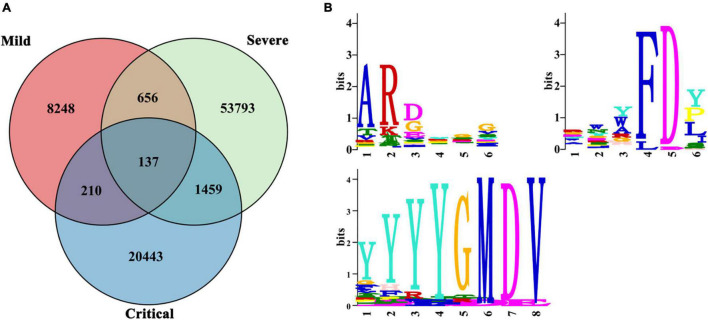
Public IGH clones. Public IGH clones among SARS-CoV-2-infected patients. **(A)** Clonal overlap among mild (*n* = 6), severe (*n* = 9), and critical (*n* = 10) patients. **(B)** Multiple EM for Motif Elicitation (MEME) motif for three common motifs: ARDYGG, RWYFDY, and YYYYGMDV. The relative size of each amino acid symbol is proportional to its frequency, while the total height of amino acid symbols indicates the information content of the position in bits.

We analyzed the amino acid sequences of all clones except for singletons of all patients. We exclude singletons because they are likely to be naïve B cells (Britanova et al., 2014), which may not be associated with the disease. The three patient groups shared 137 clonotypes. Also, patients in the severe and critical groups shared more clones (1,459) than those in the mild group (210 and 656) (Figure 6A).

Multiple EM for Motif Elicitation (MEME) analysis (Bailey et al., 2015) identified three motifs highly conserved in the 137 clonotypes of all COVID-19 patients in our study—ARDYGG (131/137 shared), RWYFDY (43/137 shared), and YYYYGMDV (31/137 shared) (Figure 6B). The relative size of each amino acid symbol was proportional to its frequency, while the total height of amino acid symbols indicated the information content of the position in bits.

## Discussion

The study provided comprehensive immunological profiles of three COVID–9 infected cohorts based on the clinical severity levels. Like other pathogen infections, coronavirus infections can induce sustained cytokine or chemokine responses, leading to immune disorders and high mortality. B lymphocyte is the main participant and an important indicator to detect the function of immunity (Wang et al., 2020). Therefore, it is of great significance to explore the characteristics of B lymphocyte and immune mechanisms in COVID-19 patients.

In our study, B lymphocyte counts in the critical group are significantly lower than in the mild group (*p* < 0.01). T lymphocyte counts in the critical and severe groups are significantly lower than those in the mild groups (*p* < 0.01). A negative correlation is shown between immune response and severity, similar to other reports (Lei et al., 2020; Luchsinger et al., 2020). The absolute number of B lymphocytes and T lymphocytes from COVID-9 patients may be closely related to the severity of the disease.

To further investigate the B-cell immune repertoires in COVID-19 patients, we examined the clonal features by quantifying the clonal expansion and diversity in the B-cell IGH repertoires. We examined the correlation between clinical severity and clonal features in the B-cell IGH repertoires. The study showed that IGH singleton frequency in patients is significantly lower than in HDs. Besides, the clonality index of IGH in patients is significantly higher than in HDs. Singleton frequency refers to the proportion of monoclonal B cells. The higher the initial proportion of monoclonal B cells, the stronger the ability to recognize strange antigens. The clonality index reflects the ability of B cells to replicate and proliferate after the stress caused by the specific recognition of the antigen. The greater the value, the more the clonality expansion. The results indicate that B cells in patients recognized SARS-CoV-2 and produced stress proliferation.

To visualize the IGH repertoire change more efficiently, we evaluated the dynamics in IGH repertoires and therapeutic effects. We focused on the changes of the top five clones’ amino acid sequences of CDR3 of IGH before and after treatment of critical patients. The radian marked with the amino acid sequence displayed the frequency of the most expanded B-cell clones. The larger radian means a higher frequency (Wang et al., 2019; Sheng et al., 2021). We found that the radian area of the top five clones became larger in the improvement group. However, the radian area was diminished in the deterioration group. The difference of IGH clonality between post- and pretreatment was significant in the two groups. It indicated that the improvement group could contain higher SARS-CoV-2-specific clonality. The top five IGH clonal change might be a potential indicator to predict therapeutic effect.

A major ongoing challenge to natural immunity and vaccination to COVID-19 is due to circulating SARS-CoV-2 mutant variants (Tan et al., 2021). Because a convergent effect might be expected when patients were infected with similar antigens, leading to highly expanded BCRs sharing the same IGH repertoire, it is necessary to understand the public clonotypes to SARS-CoV-2 virus among patients. As the pandemic continues, knowledge of public clone types would inform vaccination assessment. Our study found three common public motifs in shared clones by all COVID-19 patients—ARDYGG, RWYFDY, and YYYYGMDV—analyzed by MEME (Bailey et al., 2015). We believed that these three motifs might be related to the SARS-CoV-2 antigen. This bias suggested a potential requirement of particular CDR3 sequences to efficiently recognize SARS-CoV-2, which might be used in future vaccine development.

However, some limitations of our study should be recognized. First, the study’s sample size was not large, and this is a single-center study. Second, this dataset lacked blood samples after patients recovered.

## Conclusion

B cells are critical to producing antibodies and protective immunity to viruses. Here, we show that patients infected with SARS-CoV-2 who develop COVID-19, in the early stage, display B cells that could recognize SARS-CoV-2 and produce clonal expansion. Patients who had better outcomes after treatment had higher IGH clonality. All patients shared three common public motifs—ARDYGG, RWYFDY, and YYYYGMDV—which might be related to the SARS-CoV-2 antigen. These results indicated the potential of IGH profiling as a tool to help understand patient responses and might be used for vaccine development.

## Data Availability Statement

The original contributions presented in the study are included in the article/supplementary material, further inquiries can be directed to the corresponding authors.

## Ethics Statement

The studies involving human participants were reviewed and approved by the Ethics Committee of The First Affiliated Hospital of Zhejiang University. The patients/participants provided their written informed consent to participate in this study.

## Author Contributions

XF, HC, and TS wrote the first draft. JH, LH, CG, MX, and YG participated in the discussion. All authors cared for the patients and reviewed the final manuscript.

## Conflict of Interest

The authors declare that the research was conducted in the absence of any commercial or financial relationships that could be construed as a potential conflict of interest.

## Publisher’s Note

All claims expressed in this article are solely those of the authors and do not necessarily represent those of their affiliated organizations, or those of the publisher, the editors and the reviewers. Any product that may be evaluated in this article, or claim that may be made by its manufacturer, is not guaranteed or endorsed by the publisher.

## Footnotes

1
https://meme-suite.org/meme/
